# Supplementary material for: Clinical Nomogram Model for Pre-Operative Prediction of Microvascular Invasion of Hepatocellular Carcinoma before Hepatectomy
Source: Medicina (Kaunas). 2024 Aug 28;60(9):1410. doi: 10.3390/medicina60091410 (PMC11433876; doi:10.3390/medicina60091410)
Supplement: Supplementary file 1 [file medicina-60-01410-s001.zip › medicina-3144952-supplementary.pdf]

Supplement:

Clinical Nomogram Model for Pre-operative Prediction of Microvascular Invasion of Hepatocellular Carcinoma Before Hepatectomy

Table S1. Odds ratio of MVI were illustrated using logistic regression analysis from basic and tumor profiles

Variables	β	Wald score	OR(95%CI)	P value
Cohort				
Training set(ref.)			1	
Validation set	0.374	3.555	1.454(0.985, 2.146)	0.059
Age	0.001	0.003	1.001(0.983, 1.019)	0.956
Sex				
Male(ref.)			1	
Female	-0.191	0.628	0.826(0.515, 1.325)	0.428
DM				
non(ref.)			1	
Y es	-0.045	0.039	0.956(0.613, 1.491)	0.843
HbA1C	-0.303	3.498	0.739(0.538, 1.015)	0.061
Alc				
0(ref.)			1	
1+2	0.213	1.042	1.238(0.822, 1.864)	0.307
ECOG				
0+1(ref.)			1	
2+3+4	0.863	0.738	2.371(0.331, 16.994)	0.390
BCLC				
0+1(ref.)			1	
2+3	1.056	26.971	2.876(1.930, 4.284)	<0.001
Child-Pugh				
A(ref.)			1	
B	0.319	0.188	1.376(0.325, 5.836)	0.665
Hepatitis				
non(ref.)			1	
B/C	0.192	0.551	1.211(0.730, 2.008)	0.458
Tumor extension				
non (ref.)			1	
yes	1.277	13.702	3.586(1.824, 7.051)	<0.001
Tumor size	0.017	35.627	1.017(1.011, 1.023)	<0.001

Tumor number	0.528	4.891	1.695(1.062, 2.705)	0.027
Satellite nodule				
non (ref.)			1	
yes	1.997	52.046	7.368(4.283, 12.677)	<0.001

Table S2. Odds of MVI were illustrated using logistic regression analysis of laboratory data and nutrition-based index.

Variables	β	Wald score	OR(95%CI)	P value
INR	0.080	2.094	1.083(0.972, 1.208)	0.148
ICG	-0.008	0.537	0.992(0.970, 1.014)	0.464
AFP(×10^2)	0.001	9.842	1.001(1.000, 1.002)	0.002
WBC(×10^3)	0.073	3.519	1.075(0.997, 1.160)	0.061
Neutrophil(×10^3)	0.077	1.882	1.080(0.967, 1.207)	0.170
Lymphocyte(×10^3)	-0.003	0.000	0.997(0.729, 1.362)	0.983
Monocyte(×10^3)	~~0.633~~	~~1.051~~	~~1.883(0.561, 6.320)~~	~~0.305~~
Platelet(×10^3)	0.005	15.261	1.005(1.003, 1.008)	<0.001
Bili, mg/dl	0.003	0.000	1.003(0.594, 1.695)	0.991
GPT	0.000	0.031	1.000(0.998, 1.003)	0.861
GOT	0.002	1.981	1.002(0.999, 1.005)	0.159
GOT/GPT	0.157	3.736	1.170(0.998, 1.372)	0.053
Alb g/dl	-0.396	2.048	0.673(0.391, 1.158)	0.152
Alk.Pase	0.001	4.536	1.001(1.000, 1.002)	0.033
PNI	-0.040	2.048	0.961(0.910, 1.015)	0.152
AAR	-3.704	4.153	0.025(0.001, 0.868)	0.042
ALBI	0.326	4.598	1.386(1.028, 1.868)	0.032
GNRI	-0.020	8.272	0.981(0.967, 0.994)	0.004
